# Evaluation of CD49f as a novel surface marker to identify functional adipose‐derived mesenchymal stem cell subset

**DOI:** 10.1111/cpr.13017

**Published:** 2021-03-11

**Authors:** Kangkang Zha, Xu Li, Guangzhao Tian, Zhen Yang, Zhiqiang Sun, Yu Yang, Fu Wei, Bo Huang, Shuangpeng Jiang, Hao Li, Xiang Sui, Shuyun Liu, Quanyi Guo

**Affiliations:** ^1^ Institute of Orthopaedics Chinese PLA General Hospital Beijing Key Lab of Regenerative Medicine in Orthopaedics Key Laboratory of Musculoskeletal Trauma & War Injuries, PLA Beijing China; ^2^ School of Medicine Nankai University Tianjin China; ^3^ Musculoskeletal Research Laboratory Department of Orthopedics and Traumatology Innovative Orthopaedic Biomaterial and Drug Translational Research Laboratory Li Ka Shing Institute of Health Sciences The Chinese University of Hong Kong Hong Kong China; ^4^ The Second People’s Hospital of Guiyang Guiyang China; ^5^ Department of Orthopedics The First Affiliated Hospital of University of South China Hengyang China; ^6^ Department of Bone and Joint Surgery Affiliated Hospital of Southwest Medical University Luzhou China; ^7^ Department of Orthopedics The First Hospital of China Medical University Shenyang China

**Keywords:** adipose tissue‐derived mesenchymal stem cells, CD49f, cellular function, stem cell subset

## Abstract

**Objectives:**

CD49f is expressed on a variety of stem cells and has certain effects on their cytological functions, such as proliferation and differentiation potential. However, whether CD49f is expressed on the surface of adipose tissue‐derived mesenchymal stem cells (ADSCs) and its effect on ADSCs has not been clarified.

**Materials and methods:**

The effects of in vitro culture passage and inflammatory factor treatment on CD49f expression and the adhesion ability of ADSCs from mice and rats were investigated. CD49f^+^ cells were selected from rat ADSCs (rADSCs) by magnetic‐activated cell sorting (MACS), and the cellular functions of CD49f^+^ ADSCs and unsorted ADSCs, including their clonogenic, proliferation, adipogenic and osteogenic differentiation, migration and anti‐apoptotic capacities, were compared.

**Results:**

CD49f expression and the adhesion ability of ADSCs decreased with increasing in vitro culture passage number. TNF‐α and IFN‐γ treatment decreased CD49f expression but increased the adhesion ability of ADSCs. After CD49f was blocked with an anti‐CD49f antibody, the adhesion ability of ADSCs was decreased. No significant difference in clonogenic activity was observed between unsorted ADSCs and CD49f^+^ ADSCs. CD49f^+^ ADSCs had greater proliferation, adipogenic and osteogenic differentiation, migration and anti‐apoptotic capacities than unsorted ADSCs.

**Conclusion:**

In the current study, the expression of CD49f on ADSCs was identified for the first time. The expression of CD49f on ADSCs was influenced by in vitro culture passage number and inflammatory factor treatment. Compared with unsorted ADSCs, CD49f ^+^ ADSCs exhibited superior cellular functions, thus may have great application value in mesenchymal stem cell (MSC)‐based therapies.

## Graphical abstract

Although mesenchymal stem cells (MSCs) is one of the most promising seed cells in tissue engineering, its application faces some problems, such as an insufficient cell source, cell senescence and the loss of ‘stemness’ during in vitro culture, and rapid cell death after in vivo transplantation. To address these issues, we isolated CD49f^+^ cells from ADSCs by magnetic activated cell sorting and confirmed that CD49f^+^ ADSCs possessed greater proliferation, multiple differentiation, migration and anti‐apoptotic potential than unsorted ADSCs. After treatment with anti‐CD49f antibody, ADSCs exhibited a significantly reduced adhesion ability. These results indicated that CD49f^+^ ADSCs have superior cellular functions and considerable application prospects.

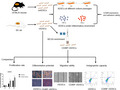

## 1 INTRODUCTION

Mesenchymal stem cells (MSCs) are multipotent stem cells originating from the bone marrow stromal cells. Due to their self‐renewal, multiple differentiation and immune regulation potential, MSCs are widely used in the treatment of various degenerative, traumatic and inflammatory diseases.^1^, ^2^, ^3^ To standardize the research and application of MSCs, the International Society for Cell Therapy provided the following minimum criteria for the definition of MSCs: (1) MSCs must be plastic‐adherent under standard culture conditions; (2) MSCs must express CD105, CD73 and CD90 and not express CD45, CD34, CD14 or CD11b; CD79 or CD19; and HLA‐DR, and (3) MSCs must have the ability to differentiate into adipocytes, osteoblasts and chondrocytes in vitro.^4^ MSCs have been found in a variety of tissues, such as adipose tissue,^5^ bone marrow,^6^ umbilical cord,^7^ synovial membrane,^8^ peripheral blood^9^ and skin,^10^ and among these MSCs, bone marrow‐derived MSCs (BMSCs) and adipose tissue‐derived MSCs (ADSCs) are most widely studied and applied. Adipose tissue consists of mature adipocytes and vascular stromal components, called stromovascular fraction (SVF). SVF contains a variety of cells, including MSCs and non‐MSCs; the SVF‐derived MSCs mainly consist of endothelial progenitor cells (CD45^−^CD31^+^CD34^+^), adventitial stromal cells (CD45^−^CD31^−^CD146^−^CD34^+^) and pericytes (CD45^−^CD31^−^CD34^−^CD146^+^).^11^ ADSCs possess greater proliferation, anti‐apoptotic and immunoregulatory abilities than BMSCs.^12^, ^13^ In addition, ADSCs have great clinical value as there is sufficient source of ADSCs that are easily acquired with little trauma.^14^ Up to 500 times more, stem cells were reported to be isolated from adipose tissue than from bone marrow.^15^

Mesenchymal stem cells from the same tissue source are also heterogeneous in their phenotypes and functions.^16^ In addition to CD73, CD90 and CD105, some novel cell surface markers have also been found to be expressed on MSCs, which can reflect the developmental status of progeny from a common precursor of MSCs during culture expansion or the physiological state of MSCs in different microenvironments. Identifying sets of unique surface markers associated with various stages of MSCs will improve MSC‐based therapies.^17^ For example, Mifune et al found that CD271^+^ BMSCs exhibited a stronger proliferation ability in vitro and more effectively promoted cartilage regeneration in rats than unsorted BMSCs.^18^ In our previous experiments, we observed that CD146^+^ ADSCs possessed stronger immunoregulatory potential than unsorted ADSCs. When injected into the cartilage defect area in animal models, CD146^+^ ADSCs were found to be more effective in reducing the early inflammatory response, resulting in a better regenerative effect.^19^

CD49f, also known as integrin α6, is a transmembrane protein that mediates cell‐cell and cell‐extracellular matrix adhesion.^20^ It is expressed on the surface of a number of stem cells, and many studies have shown that CD49f positive stem cells possess stronger ‘stemness’.^21^ For example, researchers found that CD49f could be used as a surface marker of haematopoietic stem cells (HSCs) with great potential to generate long‐term multilineage grafts, while HSCs without CD49f expression represented only transiently multipotent progenitors.^22^ In terms of MSCs, CD49f has been found to be expressed on the surfaces of BMSCs,^23^, ^24^, ^25^ dental pulp‐derived MSCs (DPSCs),^26^ skin‐derived MSCs (SSCs)^27^ and umbilical cord blood‐derived MSCs (UCBMSCs).^28^ It was reported that CD49f^+^ BMSCs possess greater colony‐forming, proliferation, migration and multiple differentiation abilities than CD49f^‐^MSCs.^23^, ^24^ Lee et al intravenously injected MSCs into mice with myocardial infarction and found that PODXL^hi^/CD49f^hi^ BMSCs were more likely to in lung and migrate to damaged cardiac and renal tissues.^29^ These findings indicate that CD49f^+^ BMSCs represent an MSC subset with ideal cellular functions and therapeutic potential. However, whether CD49f is expressed on ADSCs and the effects of CD49f on the cytological functions of ADSCs have not yet been studied. Since adipose tissue could provide a sufficient cell source for the isolation and application of MSC subpopulations, further studies focusing on the role of CD49f in ADSCs are urgently needed.

In the current study, we first examined the expression level of CD49f in ADSCs at different culture passages and the effect of inflammatory factors on CD49f expression in ADSCs. Then, we verified that CD49f plays an important role in regulating the adhesion ability of ADSCs. Finally, we isolated CD49f^+^ ADSCs by magnetic‐activated cell sorting (MACS) and found that CD49f^+^ ADSCs have stronger proliferation, adipogenic and osteogenic differentiation, migration and anti‐apoptotic capacities than unsorted ADSCs.

## 2 MATERIALS AND METHODS

### 2.1 Animals

C57BL/6N mice and Sprague‐Dawley (SD) rats were purchased from Beijing Vital River Laboratory Animal Technology Co., Ltd. All procedures were approved by the Chinese PLA General Hospital Health Sciences Institutional Review Board.

### 2.2 Isolation and culture of ADSCs

Adipose tissue taken from C57BL/6N mice and SD rats was washed with phosphate‐buffered saline (PBS, Corning, NY, USA) and minced into 1‐mm^3^ pieces. The fragmented adipose tissue was digested with 0.25% type I collagenase (Gibco, ThermoFisher Scientific, Waltham, MA, USA) in a shaker at 37°C for 40 minutes. Digestion was terminated by the addition of Dulbecco's modified Eagle's medium/F12 medium (DMEM/F12) (Gibco, ThermoFisher Scientific) culture solution containing 10% foetal bovine serum (FBS) (Gibco, ThermoFisher Scientific), and cells were centrifuged for 5 minutes at 300 ***g***. The precipitate was filtered through 100‐μm filters and washed twice with PBS. Cells were cultured in DMEM/F12 supplemented with 10% FBS in 5% CO_2_ at 37°C. The medium was changed every 2‐3 days, and the cells were passaged after reaching 80% confluence using 0.25% trypsin (Gibco, ThermoFisher Scientific).

### 2.3 MACS

CD49f^+^ cells were isolated from rat ADSCs (rADSCs) at passage 3 (P3) by MACS using PE‐conjugated anti‐CD49f antibody and magnetically labelled anti‐PE antibody. In brief, the cells were stained with a PE‐conjugated anti‐CD49f antibody (Bio‐Rad, Hercules, CA, USA) in the dark at 4°C for 30 minutes and washed with PBS. Then, the cell pellet was resuspended in 100 µL of 1% BSA containing 20 µL of anti‐PE MicroBeads (Miltenyi Biotec, Bergisch Gladbach, Germany) and incubated at 4°C for 15 minutes. A Vario MACS separator (Miltenyi Biotec) was used for magnetic separation. The magnetically labelled CD49f^+^ ADSCs were collected for further experiments.

### 2.4 ADSC differentiation

Mouse ADSCs (mADSCs) and rADSCs at P3, as well as CD49f^+^ rADSCs, were plated onto 12‐well culture plates (Falcon Corning, Tewksbury, MA, USA) at a density of 1 × 10^4^ cells/cm^2^ for osteogenesis and adipogenesis using MSC adipogenic and osteogenic culture medium (Cyagen, Santa Clara, CA, USA). Relative gene expression levels were measured by quantitative real‐time PCR (RT‐PCR) 7 days after induction. On the 14th day, cells were stained with Oil Red O to assess adipogenesis or alkaline red to assess osteogenesis.

For chondrogenic differentiation, 3 × 10^5^ mADSCs and rADSCs at P3 in pellets were induced using chondrogenic culture medium, which was consisted of High Glucose Dulbecco's Modified Eagle Medium (Gibco, ThermoFisher Scientific) supplemented with 1% (v/v) Insulin, Transferrin, Selenium Solution (Sigma Aldrich, St. Louis, MO, USA), 1% (v/v) antibiotic/antimycotic solution (Sigma Aldrich), 100 nmol/L dexamethasome (Sigma Aldrich), 50 μg/mL ascorbic acid‐2‐phosphate (Sigma Aldrich), 40 μg/mL l‐proline (Sigma Aldrich) and 10 ng/mL TGF‐β3 (Peprotech). Cell pellets were stained with Alcian blue 21 days after induction.

### 2.5 Cell treatment with TNF‐α and IFN‐γ

mADSCs and rADSCs at passage 2 (P2) were cultured at a density of 2 × 10^4^ cells/cm^2^ in DMEM F12 supplemented with 10% FBS in the absence or presence of either 40 ng/mL TNF‐α (Sigma‐Aldrich) or 20 ng/mL IFN‐γ (R&D Systems, Minneapolis, MN, USA) for 24 hours.

### 2.6 Flow cytometry

mADSCs and rADSCs at P3 were harvested by enzymatic digestion, centrifuged and washed with PBS. The cells were then stained with anti‐mouse antibodies against CD34‐APC (Biorbyt, Cambridge, Cambridgeshire, UK), CD45‐APC (BioLegend, San Diego, CA, USA), CD90‐APC (BioLegend) and SCA‐1‐APC (BioLegend) or anti‐rat antibodies against CD34‐PE (BioLegend), CD45‐PE (BioLegend), CD90‐PE (BioLegend) and CD105‐PE (Santa Cruz Biotechnology, Santa Cruz, CA, USA). mADSCs and rADSCs at passage 1 (P1)‐ passage 6 (P6) or isolated CD49f^+^ rADSCs were stained with anti‐CD49f antibody (mADSCs: Invitrogen, ThermoFisher Scientific, Waltham, MA, USA; rADSCs: Bio‐Rad). To investigate whether the expression of CD49f in ADSCs was influenced by inflammatory environment, ADSCs at P2 were treated with 40 ng/mL TNF‐α (Sigma‐Aldrich) or 20 ng/mL IFN‐γ (R&D Systems) for 24 hours and then stained with anti‐CD49f antibody (mADSCs: Invitrogen, ThermoFisher Scientific; rADSCs: Bio‐Rad) and anti‐VCAM‐1 antibody (mADSCs: BioLegend; rADSCs: BioLegend).

The cells were kept in the dark for 30 minutes and then washed twice with PBS. Cytometric analysis was performed using a BD FACSCelesta™ flow cytometer (Becton Dickinson, Franklin Lakes, NJ, USA), and the results were analysed using FlowJo software.

### 2.7 Immunofluorescence

mADSCs and rADSCs at P2 and P6 were fixed with 4% paraformaldehyde (PFA) for 10 minutes at room temperature, washed with PBS, permeabilized with 0.25% Triton X‐100 for 10 minutes and blocked with 10% goat serum for 30 minutes. To verify the effect of inflammatory environment on expression of CD49f in ADSCs, mADSCs and rADSCs at P2 were treated with 40 ng/mL TNF‐α (Sigma‐Aldrich) or 20 ng/mL IFN‐γ (R&D Systems) for 24 hours before cell fixation. Then, the cells were incubated with an anti‐CD49f antibody (mADSCs: Invitrogen, ThermoFisher Scientific; rADSCs: Bio‐Rad). The next day, the cells were washed and incubated with a secondary antibody in the dark for 1 hour at room temperature. Cells were counterstained with DAPI diluted 1:500 for 3 minutes and viewed with an Olympus IX70 microscope (Olympus, Tokyo, Japan). Images were captured with an Olympus DP71 CCD camera (Olympus).

### 2.8 Adhesion assay

A 96‐microwell plate (Falcon Corning) was coated with 10 mg/mL laminin (Sigma‐Aldrich) overnight at 4°C and then blocked with 1% bovine serum albumin (BSA) (Sigma‐Aldrich). mADSCs and rADSCs at P2 and P6 were seeded in quadruplicate at 2.5 × 10^4^ cells/well and incubated for 30 minutes at 37°C. To investigate whether the adhesion of ADSCs to laminin was mediated by CD49f, ADSCs at P3 were treated with anti‐CD49f antibody (mADSCs: Invitrogen, ThermoFisher Scientific; rADSCs: Bio‐Rad) or isotype control antibody (mADSCs: Becton Dickinson; rADSCs: Bio‐Rad) for 30 minutes before seeding on the plate at 2.5 × 10^4^ cells/well. To investigate the effect of inflammatory environment on ADSCs adhesion ability, mADSCs and rADSCs at P2 were treated with 40 ng/mL TNF‐α (Sigma‐Aldrich) or 20 ng/mL IFN‐γ (R&D Systems) for 24 hours before seeding on the plate at 1 × 10^4^ cells/well. The medium was removed, and the plate was washed with PBS 3 times. Adherent cells were stained with 0.1% crystal violet and scanned with an Olympus BH‐2 light microscope (Olympus). Images were captured by using an Olympus DP71 CCD camera (Olympus). Adherent cells in 4 randomly chosen fields were counted. Each experiment was performed in triplicate.

### 2.9 Colony‐forming unit fibroblastic (CFU‐F) assay

CD49f^+^ rADSCs and unsorted rADSCs at P3 were seeded onto a 6‐well plate (Falcon Coring) at 200 cells/well and cultured in DMEM/F12 supplemented with 10% FBS in 5% CO_2_ at 37°C. Culture medium was changed every 3‐4 days. Cells were fixed with 4% PFA and stained with 0.1% crystal violet on day 14. Colonies larger than 50 cells were counted.

### 2.10 Cell cycle assay

CD49f^+^ rADSCs and unsorted rADSCs at P3 were fixed in 70% ethanol for 2 hours and washed with PBS. Cell pellets were incubated with RNase A and propidium iodide (PI) (Beyotime, Beijing, China) at 37°C in the dark for 30 minutes. Cytometric analysis was performed using a BD FACSCelesta™ flow cytometer (Becton Dickinson), and the results were analysed using ModiFit software.

### 2.11 Evaluation of proliferation by the Cell Counting Kit‐8 (CCK‐8) assay

CD49f^+^ rADSCs and unsorted rADSCs at P3 were seeded onto a 96‐well plate (Falcon Coring) at 2 × 10^3^ cells/well and cultured in DMEM/F12 supplemented with 10% FBS in 5% CO_2_ at 37°C. Ten microliters of CCK‐8 reagent (Dojindo, Kumamoto, Japan) was added after 1, 3 and 5 days of culture. After incubation at 37°C for 2.5 hours, the optical density (OD) value (absorbance at 450 nm) was measured with an EPOCH microplate reader (BioTek, Winooski, VT, USA). The cell proliferation (fold change) was calculated as follows:

Cell proliferation(fold change)=(Ax‐Ab)/(A1‐Ab).

where *A_x_* represents the average OD values of cells at day 1, 3 or 5; *A_b_* represent the average OD values of control plate (without cells); *A*_1_ represents the average OD values of cells at day 1.

### 2.12 Migration assay

CD49f^+^ rADSCs and unsorted rADSCs at P3 were seeded in a 24‐well plate (Falcon Corning) at 1 × 10^5^ cells/well and cultured in DMEM/F12 containing 10% FBS medium. After reaching 90% confluence, a bidirectional wound on the monolayer cells was formed with a sterile pipette tip, and the cell debris was washed away with PBS. Then, DMEM/F12 containing 1% FBS was added, and the cells were incubated in 5% CO_2_ at 37°C. Wound closure was measured at 0, 12 and 24 hours after wounding. Each experiment was performed in triplicate.

### 2.13 Apoptosis assay

CD49f^+^ rADSCs and unsorted rADSCs at P3 were seeded in 6‐well plates (Falcon Corning) at 2 × 10^5^ cells/well and treated with 10 ng/mL IL‐β (Abcam, Cambridge, Cambridgeshire, UK) for 24 hours. Afterwards, the cells were washed with PBS and digested with trypsin. They were then resuspended in binding solution, and Annexin V‐FITC and PI (Solarbio, Beijing, China) were added. After 20 minutes of incubation at room temperature, the cells were immediately analysed using a BD FACSCelesta™ flow cytometer (Becton Dickinson).

### 2.14 RT‐PCR

Total RNA was isolated from cells using TRIzol reagent (Invitrogen, ThermoFisher Scientific). cDNA was synthesized by reverse transcription using the FastKing RT Kit (TIANGEN, Beijing, China) for first‐strand synthesis. RT‐PCR was performed using Power SYBR Green PCR Master Mix (Applied Biosystems, ThermoFisher Scientific, Waltham, MA, USA) and a Roche LightCycler 480 real‐time PCR system (Roche, Basel, Basel‐Stadt, Switzerland). The PCR cycling conditions consisted of an initial denaturation step at 95°C for 10 minutes followed by 40 cycles of 95°C for 15 seconds and 59°C for 60 minutes. The following primers were used: PPARY: F‐TGTTATGGGTGAAACTCTGGG, R‐AGAGCTGATTCCGAAGTTGG; OCN: F‐CAGACCTAGCAGACACCATG, R‐GCTTGGACATGAAGGCTTTG; AND RUNX2: F‐CCGGGAATGATGAGAACTA, R‐GACCGTCCACTGTCACTTT.

### 2.15 Statistical analyses

The statistical analysis software SPSS 25.0 was used to analyse the differences in data from each group, and *P* < .05 indicated a significant difference. Student's *t* test or one‐way analysis of variance (ANOVA) was used for comparisons of groups.

## 3 RESULTS

### 3.1 Identification of mADSCs and rADSCs

**FIGURE 1:**
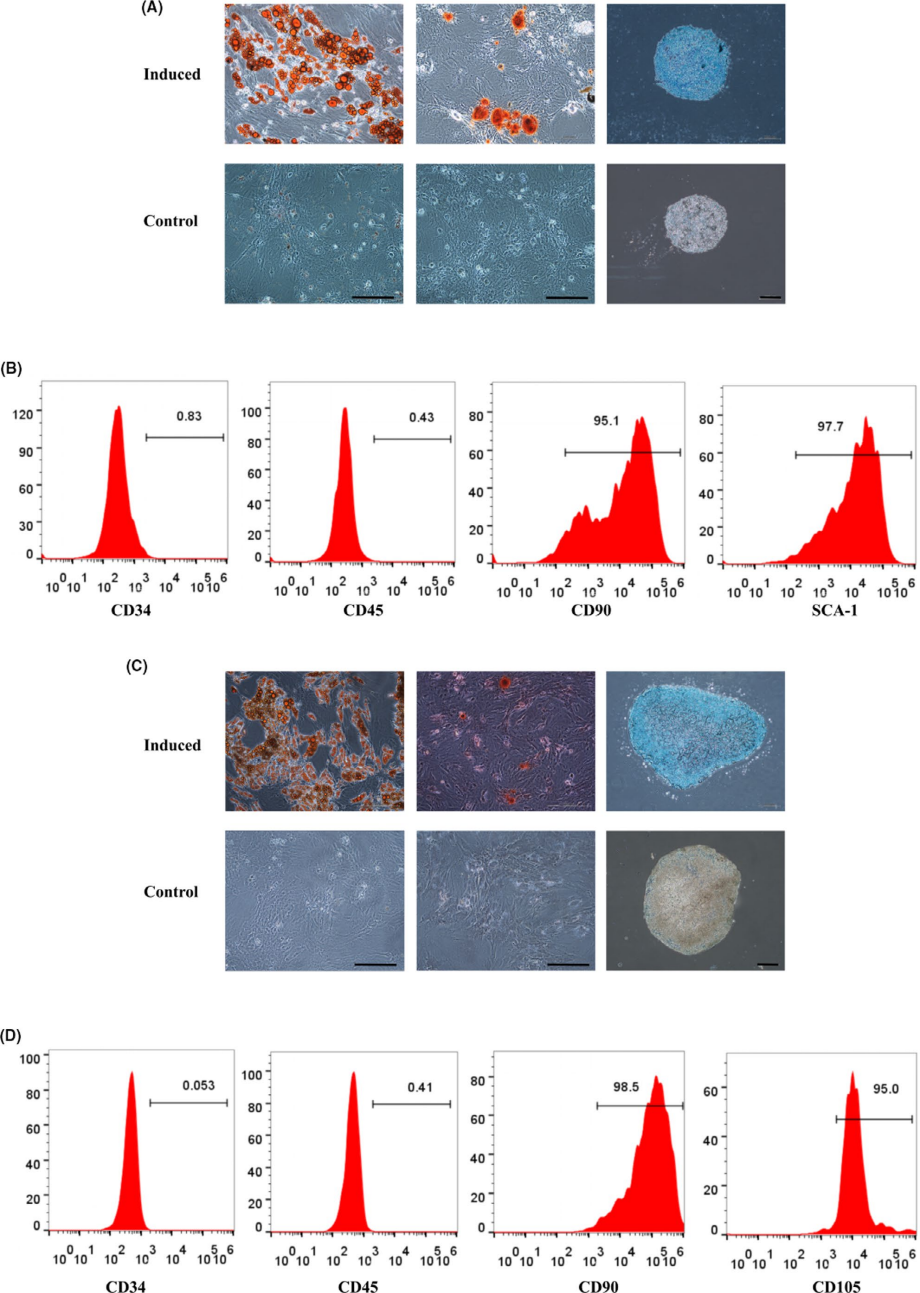
Identification of cultured mADSCs and rADSCs. A, Trilineage differentiation of mADSCs at P3 in vitro. Left: Oil Red O staining; middle: Alizarin red staining; right: Alcian blue staining. B, Flow cytometry analysis of the surface antigens of mADSCs at P3. C, Trilineage differentiation of rADSCs at P3 in vitro. Left: Oil Red O staining; middle: Alizarin red staining; right: Alcian blue staining. D, Flow cytometry analysis of the surface antigens of rADSCs at P3. Scale bar: 200 μm

Trilineage differentiation was induced to examine the multipotency of mADSCs and rADSCs. After adipogenic or osteogenic induction for 14 days, both the mADSCs and rADSCs exhibited positive Oil Red O and Alizarin red staining. After chondrogenic induction for 21 days, both mADSCs and rADSCs showed positive Alcian blue staining (Figure 1A and C). These results revealed that the cultured mADSCs and rADSCs could differentiate into adipocytes, osteoblasts and chondrocytes in vitro.

Flow cytometry analysis was performed to detect surface phenotypes of mADSCs and rADSCs at P3. mADSCs were negative for CD34 and CD45 and positive for CD90 and SCA‐1 (Figure 1B), while rADSCs were negative for CD34 and CD45 and positive for CD90 and CD105 (Figure 1D).

### 3.2 Expression of CD49f on mADSCs and rADSCs at different passages

**FIGURE 2:**
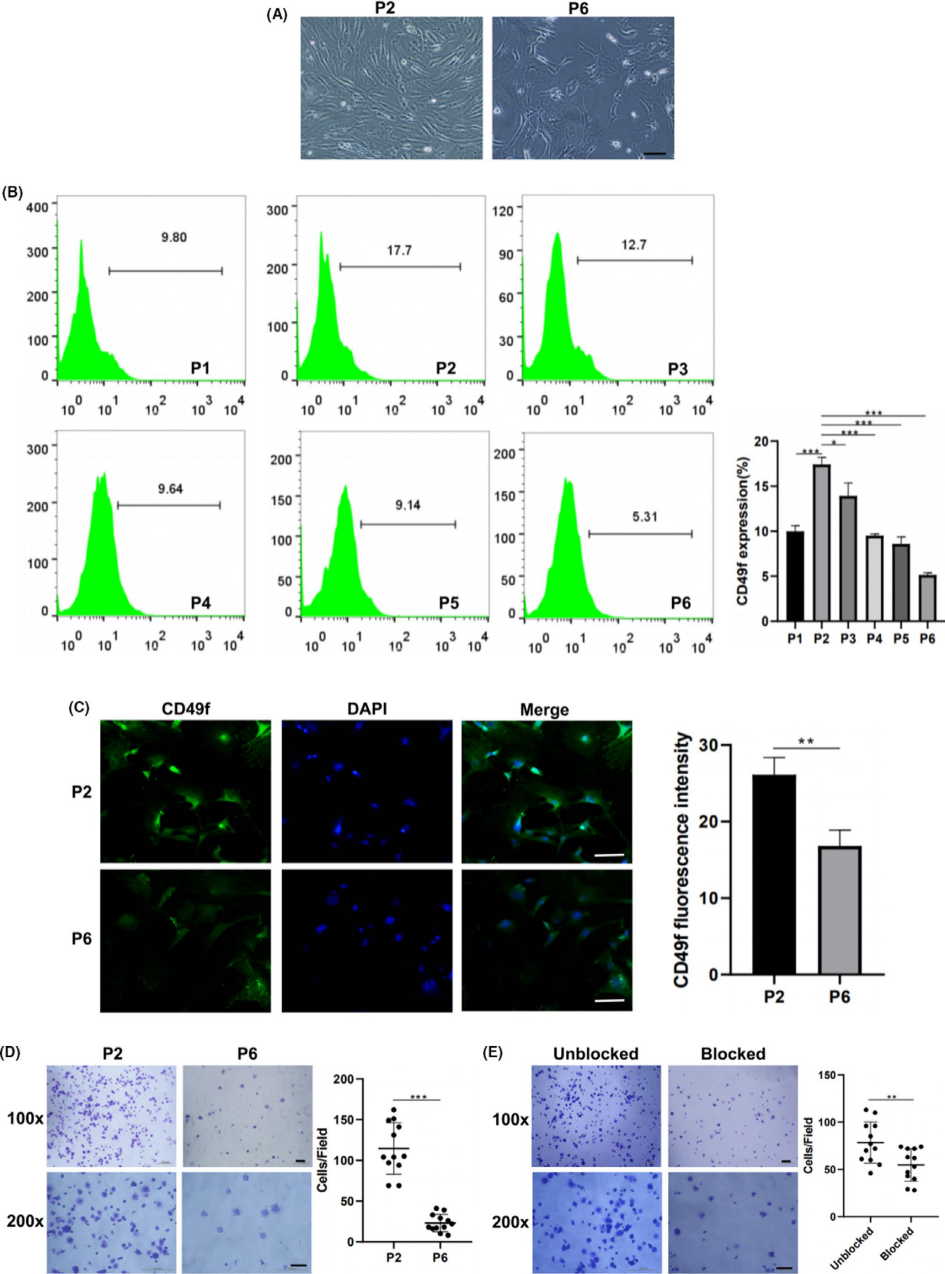
Expression of CD49f on mADSCs at different passage numbers. A, Representative morphology of mADSCs at P2 and P6. B, Flow cytometry analysis of CD49f expression on mADSCs at P1‐P6. C, Immunofluorescence images showing CD49f expression on mADSCs at P2 and P6. D, Assay of mADSCs adhesion to laminin at P2 and P6. E, Assay of mADSCs adhesion to laminin at P3 after blocking CD49f with a monoclonal antibody. Data are expressed as the mean ± standard deviation (SD), *P < .05, **P < .01, ***P < .001. Scale bar: 100 μm

**FIGURE 3:**
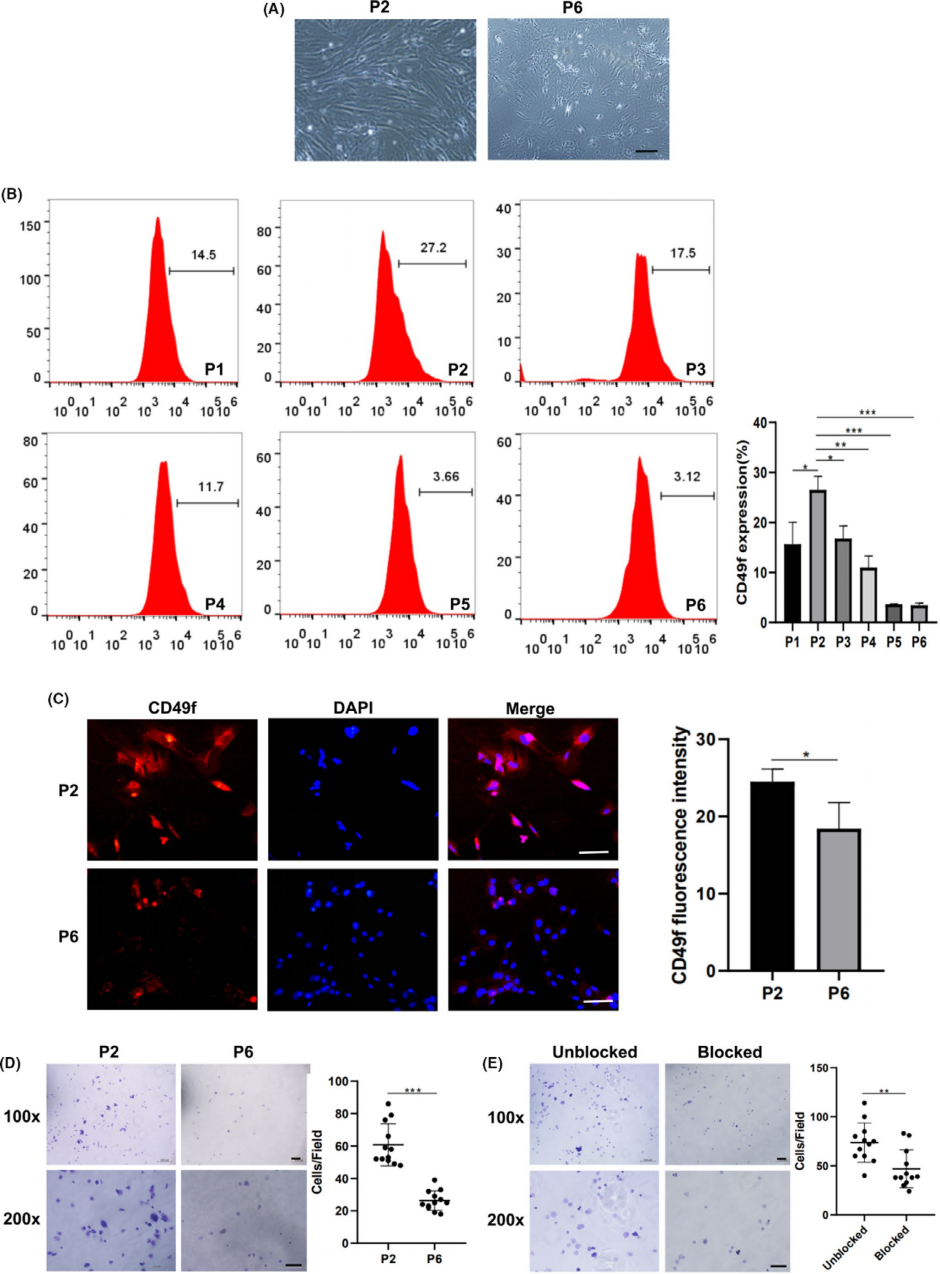
Expression of CD49f on rADSCs at different passage numbers. A, Representative morphology of rADSCs at P2 and P6. B, Flow cytometry analysis of CD49f expression on rADSCs at P1‐P6. C, Immunofluorescence images showing CD49f expression on rADSCs at P2 and P6. D, Assay of rADSCs adhesion to laminin at P2 and P6. E, Assay of rADSCs adhesion to laminin at P3 after blocking CD49f with a monoclonal antibody. Data are expressed as the mean ± SD, *P < .05, **P < .01, ***P < .001. Scale bar: 100 μm

Both mADSCs and rADSCs exhibited a fibroblast‐like morphology at P2 but became larger and less confluent and showed fewer signs of senescence at P6 (Figures 2, 3 and 2, 3). CD49f expression on mADSCs and rADSCs at different passages was determined by flow cytometry. The expression of CD49f peaked at P2 and then gradually decreased with increasing numbers of culture passages in both mADSCs (Figure 2B) and rADSCs (Figure 3B). The expression of CD49f on rADSCs was higher than that on mADSCs. Immunofluorescence staining verified that the expression of CD49f on both mADSCs and rADSCs at P6 was significantly lower than that at P2 (*P* < .05) (Figures 2, 3 and 2, 3). As shown by the adhesion assay, the adhesion of both mADSCs and rADSCs to laminin was significantly lower at P6 than P2 (*P* < .001) (Figures 2, 3 and 2, 3). An anti‐CD49f antibody was applied to investigate whether the adhesion of ADSCs to laminin was mediated by CD49f. After treatment with a monoclonal antibody against CD49f, the adhesion of both mADSCs and rADSCs to laminin was significantly inhibited (*P* < .01) (Figures 2, 3 and 2, 3).

### 3.3 Expression of CD49f on mADSCs and rADSCs after treatment with TNF‐α and IFN‐γ

**FIGURE 4:**
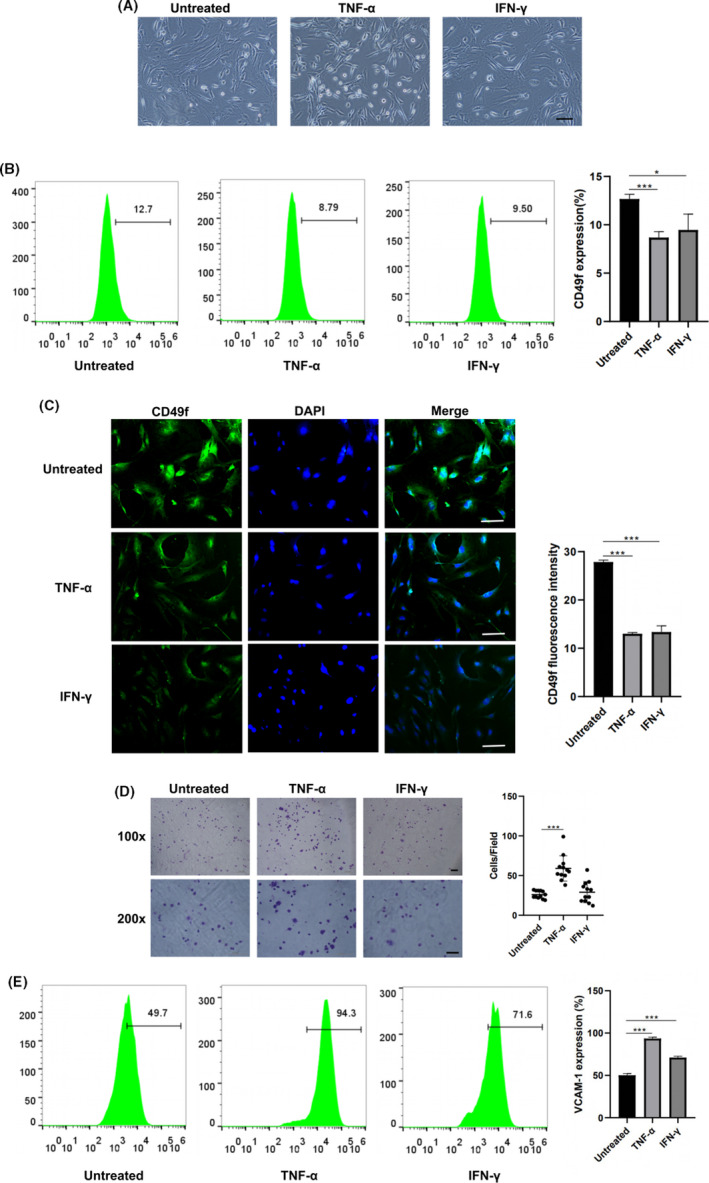
Expression of CD49f on mADSCs at P2 after treatment with TNF‐α and IFN‐γ. A, Representative morphology of mADSCs after treatment with TNF‐α and IFN‐γ. B, Flow cytometry analysis of CD49f expression on mADSCs after treatment with TNF‐α and IFN‐γ. C, Immunofluorescence images showing CD49f expression on mADSCs after treatment with TNF‐α and IFN‐γ. D, Assay of mADSCs adhesion to laminin after treatment with TNF‐α and IFN‐γ. E, Flow cytometry analysis of VCAM‐1 expression on mADSCs after treatment with TNF‐α and IFN‐γ. Data are expressed as the mean ± SD, *P < .05, ***P < .001. Scale bar: 100 μm

**FIGURE 5:**
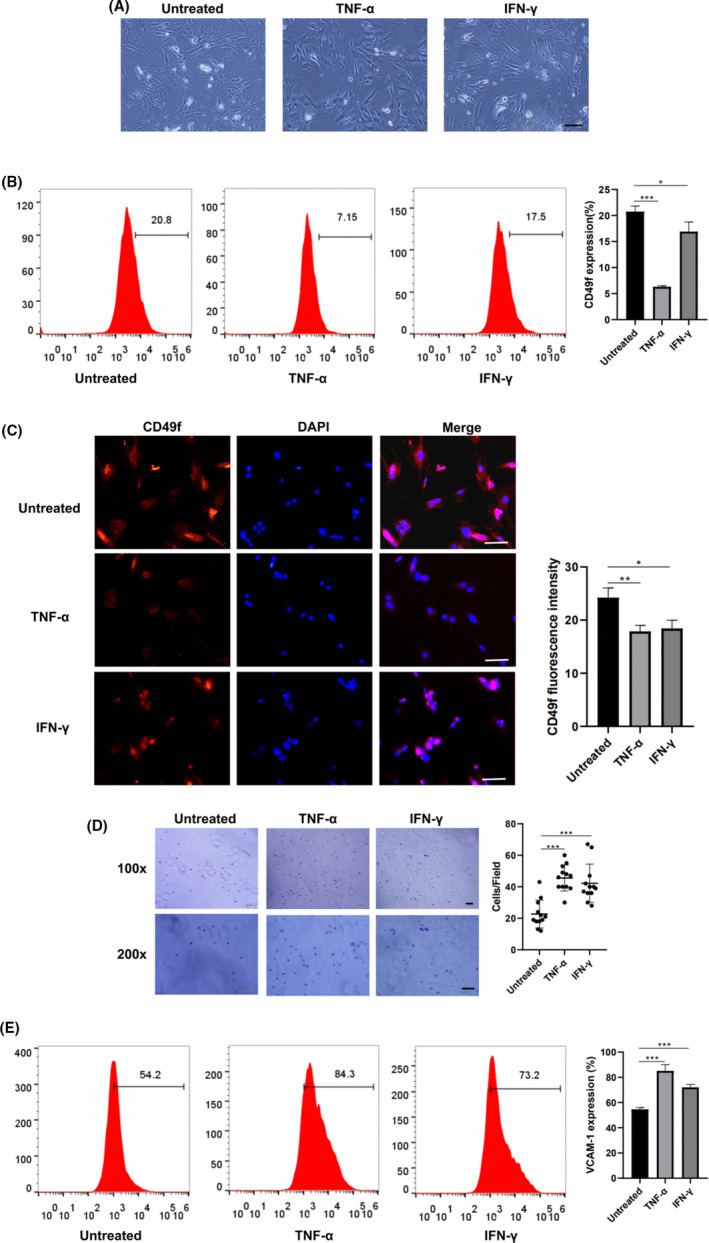
Expression of CD49f on rADSCs at P2 after treatment with TNF‐α and IFN‐γ. A, Representative morphology of mADSCs after treatment with TNF‐α and IFN‐γ. B, Flow cytometry analysis of CD49f expression on rADSCs after treatment with TNF‐α and IFN‐γ. C, Immunofluorescence images showing CD49f expression on rADSCs after treatment with TNF‐α and IFN‐γ. D, Assay of rADSCs adhesion to laminin after treatment with TNF‐α and IFN‐γ. E, Flow cytometry analysis of VCAM‐1 expression on rADSCs after treatment with TNF‐α and IFN‐γ. Data are expressed as the mean ± SD, *P < .05, **P < .01, ***P < .001. Scale bar: 100 μm

CD49f expression on mADSCs and rADSCs after treatment with TNF‐α and IFN‐γ was determined by flow cytometry. No significant difference in morphology was observed between TNF‐α‐ and IFN‐γ‐treated ADSCs and untreated ADSCs (Figures 4, 5 and 4, 5). The results of flow cytometry analysis demonstrated that after treatment with TNF‐α and IFN‐γ, the expression of CD49f was decreased on both mADSCs (mean, 12.7% vs 8.7% and 12.7% vs 9.5%; *P* < .05) (Figure 4B) and rADSCs (mean, 20.8% vs 6.3% and 20.8% vs 16.9%; *P* < .05) (Figure 5B). Immunofluorescence staining verified that the expression of CD49f on both mADSCs and rADSCs after treatment with TNF‐α and IFN‐γ was significantly lower than that on untreated mADSCs and rADSCs, respectively (*P* < .05) (Figures 4, 5 and 4, 5). As shown by the adhesion assay, the adhesion of mADSCs to laminins was significantly increased after treatment with TNF‐α (*P* < .001). No significant difference in adhesion to laminins was observed between untreated mADSCs‐ and IFN‐γ‐treated mADSCs (Figure 4D). The adhesion of rADSCs to laminins was significantly increased after treatment with TNF‐α and IFN‐γ (*P* < .001) (Figure 5D), which might be attributed to the increased expression of other adhesion‐related molecules. VCAM‐1 is a well‐known cell adhesion molecule, and we found that after treatment with TNF‐α and IFN‐γ, the expression of VCAM‐1 was significantly up‐regulated on both mADSCs (mean, 50.1% vs 93.5% and 50.1% vs 71%; *P* < .001) (Figure 4E) and rADSCs (mean, 54.6% vs 85.1% and 54.6% vs 72%; *P* < .001) (Figure 5E).

### 3.4 CD49f^+^ ADSCs exhibited greater proliferation and adipogenic and osteogenic differentiation abilities

**FIGURE 6:**
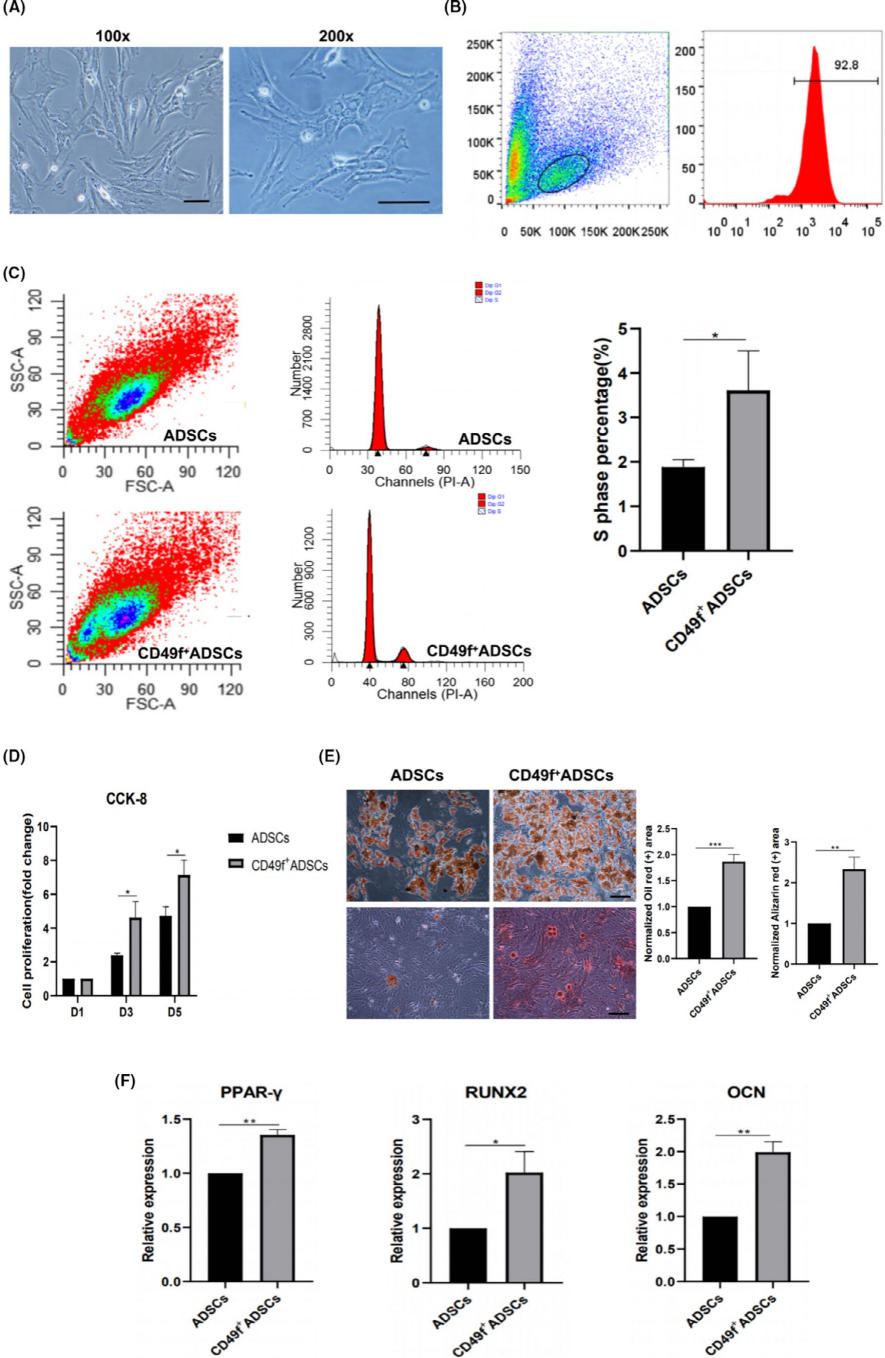
CD49f^+^ ADSCs exhibited higher proliferation and multiple differentiation abilities than unsorted ADSCs at P3. A, Morphology of CD49f^+^ ADSCs: the same culture at two different magnifications. B, Flow cytometry analysis of the positive rate of CD49f^+^ ADSC after enrichment. C, Cell cycle analysis of CD49f^+^ ADSCs and unsorted ADSCs. D, CCK‐8 assay of CD49f^+^ ADSCs and unsorted ADSCs. The cell proliferation (fold change) was calculated as follows: Cell proliferation(fold change)=(Ax‐Ab)/(A1‐Ab), where *A_x_* represents the average OD values of cells at day 1, 3 or 5; *A_b_* represents the average OD values of control plate (without cells); *A*_1_ represents the average OD values of cells at day 1. E, Adipogenic and osteogenic differentiation of CD49f^+^ ADSCs and unsorted ADSCs. Left: Oil Red O staining; right: Alizarin red staining. F, Relative mRNA expression of an adipogenic marker (PPAR‐γ) at day 7 after adipogenic induction and osteoblast markers (OCN and Runx2) at day 7 after osteogenic induction in CD49f^+^ ADSCs and unsorted ADSCs. Data are expressed as the mean ± SD, *P < .05, **P < .01, ***P < .001. Scale bar: 100 μm

To investigate the broader role of CD49f in the regulation of ADSC functions, MACS was performed to isolate CD49f^+^ subpopulations from rADSCs (Figure 6A). The positive rate of CD49f^+^ ADSCs after enrichment was examined by flow cytometry and reached 92.9% (Figure 6B). The clonogenic activity of MSCs was measured by CFU‐F assay, and the results showed that the total number of colonies formed by unsorted ADSCs and CD49f^+^ ADSCs at day 14 was not significantly different (Figure S1). The results of cell cycle assay showed that more CD49f^+^ ADSCs than unsorted ADSCs were in S phase (mean, 3.6% vs 1.9%; *P* <.05) (Figure 6C). In cell cycle, S phase plays a vital role in DNA replication. To further verify the cell cycle assay results, we performed a CCK‐8 assay and found that CD49f^+^ ADSCs grew faster than unsorted ADSCs, indicating that CD49f^+^ ADSCs possess a higher proliferation ability (*P* < .05) (Figure 6D). As shown in Figure 6E, CD49f^+^ ADSCs were more prone to differentiate into adipocytes (*P* < .001) and osteoblasts (*P* < .01) than unsorted ADSCs. After induction, the expression of the PPAR‐y (*P* < .01), RUNX2 (*P* < .05) and OCN (*P* < .01) genes in CD49f^+^ ADSCs was significantly higher than that in unsorted ADSCs (Figure 6F).

### 3.5 CD49f^+^ ADSCs exhibited higher migration and anti‐apoptotic abilities

**FIGURE 7:**
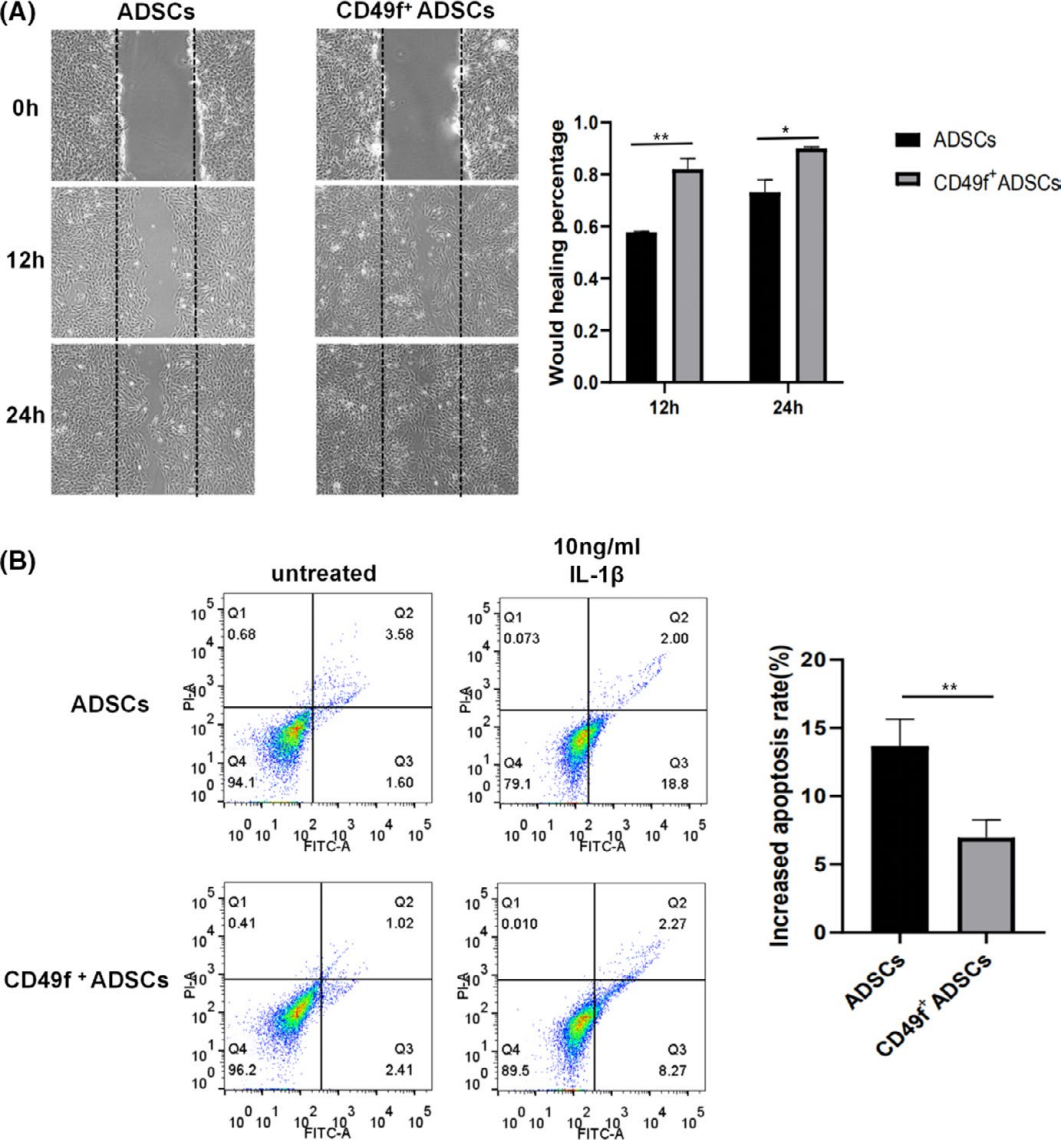
CD49f^+^ ADSCs exhibited higher migration and anti‐apoptotic abilities than unsorted ADSCs at P3. A, Wound‐healing assay of CD49f^+^ ADSCs and unsorted ADSCs. B, Apoptosis assay of CD49f^+^ ADSCs and unsorted ADSCs after treatment with 10 ng/mL IL‐β for 24 h. Data are expressed as the mean ± SD, *P < .05, **P < .01

To explore the migration ability of CD49f^+^ ADSCs, we performed a bidirectional wound‐healing assay. Both CD49f^+^ ADSCs and unsorted ADSCs gradually migrated towards the wound area over 24 hours. The mean wound‐healing percentage was 81.9% at 12 hours and 90.1% at 24 hours in CD49f^+^ ADSCs compared with 57.8% at 12 hours and 73.2% at 24 hours in unsorted ADSCs (*P* < .05) (Figure 7A). To study the anti‐apoptotic capacity of CD49f^+^ ADSCs, we treated cells with 10 ng/mL IL‐β for 24 hours to induce their apoptosis. Flow cytometry results showed that both apoptotic CD49f^+^ ADSCs and unsorted ADSCs were increased. The mean increased proportion of apoptotic cells in CD49f^+^ ADSCs was 7%, while 13.7% in unsorted ADSCs, reaching statistical significance (*P* < .01) (Figure 7B).

## 4 DISCUSSION

Mesenchymal stem cells are regarded as one of the most promising cell types in the field of tissue engineering, and their safety and efficacy have been verified.^30^ However, the application of MSCs also faces some problems, such as insufficient cell sources, cell senescence and the loss of ‘stemness’ during in vitro culture, and rapid cell death after in vivo transplantation.^31^ Besides, increasing studies have shown that MSCs are a heterogeneous population, which pose an obstacle to its clinical application.^32^, ^33^ How to reduce MSC heterogeneity is still a bottleneck and has not been effectively solved. To address these issues, we isolated CD49f^+^ cells from ADSCs by MACS and confirmed that CD49f^+^ ADSCs possessed greater proliferation, adipogenic and osteogenic differentiation, migration and anti‐apoptotic potential than unsorted ADSCs. After treatment with an anti‐CD49f antibody, ADSCs exhibited significantly reduced adhesion ability. To our knowledge, this is the first time it has been demonstrated that CD49f ^+^ ADSCs have cellular functions superior to those of unsorted ADSCs and considerable application prospects. Previous studies have confirmed that CD49f^+^ BMSCs possessed greater clonogenic capacity than CD49f^−^ BMSCs.^24^ Our data showed that CD49f^+^ ADSCs shared similar clonogenic capacity with unsorted ADSCs. In our next study, we would like to directly compare the clonogenic activities of CD49f^+^ ADSCs and CD49f^−^ ADSCs. In addition, because it was verified that ADSCs decreased their CD49f expression during the in vitro culture expansion, further studies using knock‐out and knock‐in models might help to clarify whether CD49f has impact on ADSCs colony‐forming potential.

As a transmembrane glycoprotein, CD49f is more than an adhesion molecule. It is reportedly involved in multiple signalling pathways. In fact, CD49f has two isoforms, namely, isoform A and isoform B. Although both of them are expressed in MSCs, their cytoplasmic domains are different. Yang et al claimed that the differentiation ability of BMSCs was more significantly improved by overexpression of isoform A than overexpression of isoform B.^24^ However, the role of the CD49f isoforms in ADSCs remains unclear and needs further study. The underlying signalling pathways through which CD49f modulates MSC functions have been partially elucidated. It was reported that CD49f regulated the proliferation and differentiation potentials of MSCs through activation of the PI3K/AKT/GSK3b signalling pathway and suppression of the cell cycle inhibitor proteins p53 and p21. OCT4 and SOX2, two pluripotency marker genes, could directly bind the CD49f promoter region and regulate CD49f transcription.^23^, ^34^ However, how CD49f affects the migration and anti‐apoptotic abilities of MSCs have not been uncovered. Additional signalling pathways involved in the modulation of MSC functions by CD49f remain to be explored.

In addition to proliferating and differentiating into target cells, MSCs have also been proposed to enhance tissue regeneration through paracrine actions.^35^ In response to the environment of the injured area, MSCs are activated and secrete a wide range of paracrine factors, including growth factors, cytokines, chemokines and extracellular vesicles. These factors are involved in various biological functions, such as promoting progenitor cell proliferation and differentiation, chemoattraction, immunomodulation and angiogenesis.^15^ Although CD49f^+^ ADSCs showed remarkable superiority to unsorted ADSCs in terms of their proliferation, mutidifferentiation, adhesion, migration and anti‐apoptotic abilities, investigation of their paracrine activity was absent in our research, and such an investigation will be carried out in our next study. The effect of CD49f^+^ ADSCs on other types of cells is also worth exploring. Recently, it was reported that CD49f^high^ skin‐derived MSCs were able to regulate hair follicle development of co‐cultured hair follicle epithelial cells through Notch signalling pathway, indicating that CD49f^+^ MSCs might play a role in maintaining tissue homeostasis and regeneration.^27^

In this study, we found that with increasing in vitro culture passages, the percentage of CD49f^+^ ADSCs was gradually decreased, which may partly explain the weakened adhesion ability of ADSCs after culture expansion. In addition, the expression of CD49f was susceptible to the inflammatory environment caused by TNF‐α or IFN‐γ treatment. ADSCs decreased their CD49f expression but increased their adhesion capacity after treated with TNF‐α or IFN‐γ, which may be attributed to upregulation of other adhesion molecules such as intercellular adhesion molecule‐1 (ICAM‐1) and vascular cell adhesion molecule‐1 (VCAM‐1).^36^, ^37^ We confirmed that the expression of VCAM‐1 was significantly up‐regulated in ADSCs treated with TNF‐α or IFN‐γ. Based on these results, the expression of CD49f on ADSCs seems to be inconstant, which may impede the application of CD49f^+^ subpopulations. Developments in culture conditions and bioactive molecules or materials that can maintain MSC phenotypes may be conducive to promoting the application of CD49f^+^ ADSCs in tissue engineering.

In recent years, the use of functional MSCs subset has become an important aspect of improving the therapeutic effect of MSC‐based therapy.^38^, ^39^ Since CD49f^+^ ADSCs have shown superiority over unsorted ADSCs in terms of some cellular properties, their use to repair damaged tissues and organs is quite feasible. However, to date, relevant research that verifies the effectiveness of the CD49f^+^ MSC subset in animal models is lacking. More research should be performed to address this point. A weakness of our research is that all of the experiments were performed in mADSCs and rADSCs rather than human ADSCs. The reason is that we would like to utilize CD49f^+^ ADSCs to repair tissue damage in mice and rats to verify its safety and effectiveness in vivo in our future study. It would be conducive to promoting the application of CD49f^+^ ADSCs in regenerative medicine. On the other hand, investigation on the expression and role of CD49f in human ADSCs is also quite essential, which will be conducted in our next study.

## 5 CONCLUSION

The application of MSCs faces some problems, such as insufficient cell sources, cell senescence and the loss of ‘stemness’ during in vitro culture, and rapid cell death after in vivo transplantation. CD49f has been reported to be expressed on a variety of stem cells and has certain effects on their cytological functions. We identified the expression of CD49f on ADSCs for the first time and confirmed that it was influenced by in vitro culture passage number and inflammatory factor treatment. CD49f^+^ ADSCs have shown greater adhesion, proliferation, adipogenic and osteogenic differentiation, migration and anti‐apoptotic potential than unsorted ADSCs. These results indicated that CD49f^+^ ADSCs have superior cellular functions and considerable application prospects.

## CONFLICTS OF INTEREST

The authors declare no conflicts of interest.

## **AUTHOR** **CONTRIBUTIONS**

Kangkang Zha involved in conception, experiments, data analysis, interpretation and manuscript writing; Xu Li involved in conception, design and data analysis; Guangzhao Tian, Zhen Yang and Zhiqiang Sun involved in data curation, interpretation and contribution to experiments; Yu Yang, Fu Wei, Bo Huang and Shuangpeng Jiang involved in data collection and contribution to experiments; Hao Li and Xiang Sui involved in manuscript editing and financial support; Shuyun Liu and Quanyi Guo involved in conception and design, supervision, financial support and final approval of the manuscript.

## Supporting information

Figure S1

## Data Availability

The authors confirm that the data supporting the findings of this study are available within the article and/or its Supplementary Materials.
