# Supplementary figures and images for: Evaluation of CD49f as a novel surface marker to identify functional adipose‐derived mesenchymal stem cell subset
Source: Cell Prolif. 2021 Mar 11;54(5):e13017. doi: 10.1111/cpr.13017 (PMC8088464; doi:10.1111/cpr.13017)

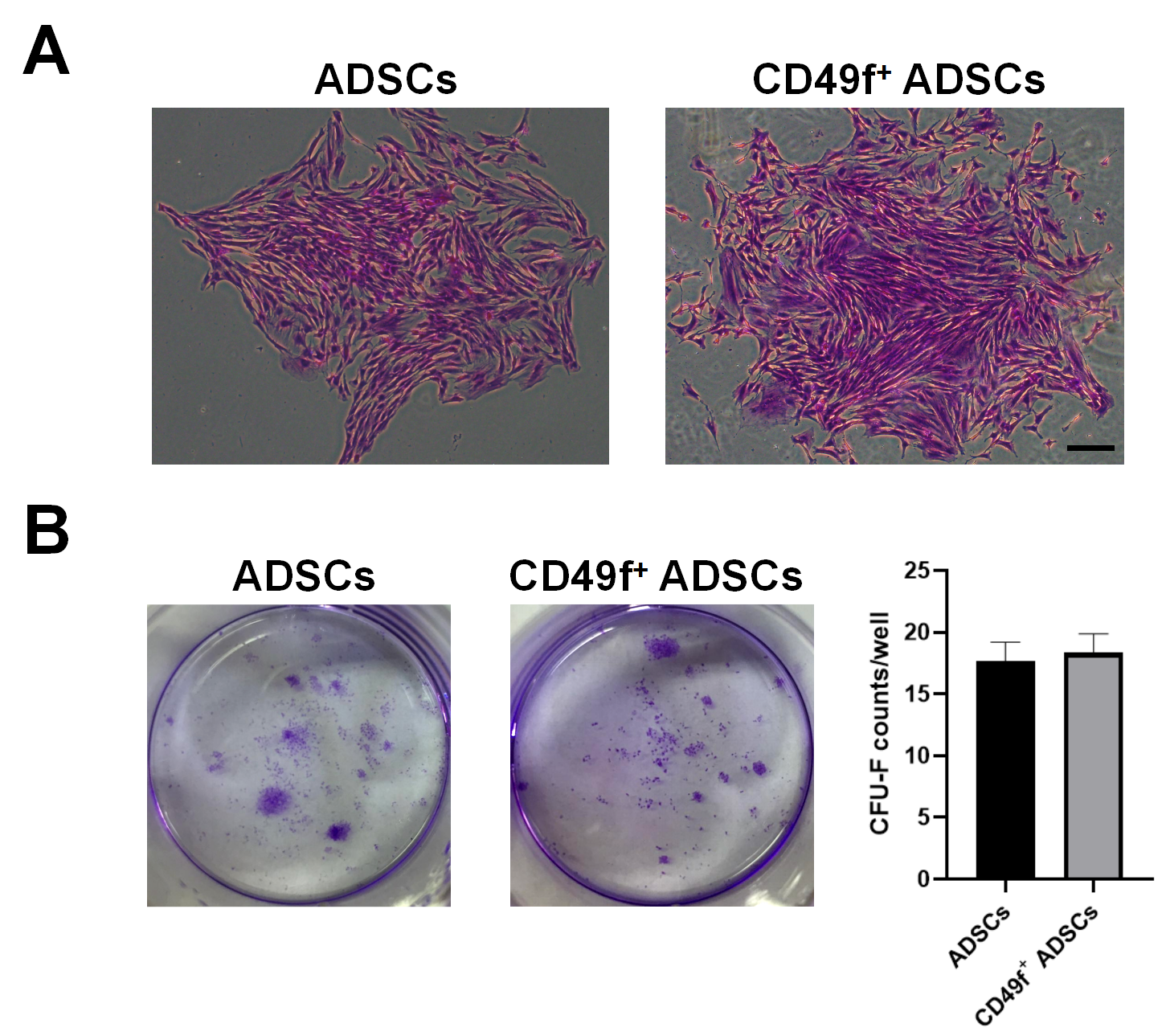

Supplement: Supplementary file 1 — Figure S1 [file CPR-54-e13017-s001.png]
